# Fluid Properties Extraction in Confined Nanochannels with Molecular Dynamics and Symbolic Regression Methods

**DOI:** 10.3390/mi14071446

**Published:** 2023-07-19

**Authors:** Dimitrios Angelis, Filippos Sofos, Konstantinos Papastamatiou, Theodoros E. Karakasidis

**Affiliations:** Condensed Matter Physics Laboratory, Department of Physics, University of Thessaly, 35100 Lamia, Greece; dimangelis@uth.gr (D.A.); kpapmath@gmail.com (K.P.); thkarak@uth.gr (T.E.K.)

**Keywords:** nanochannels, molecular dynamics, diffusion coefficient, shear viscosity, thermal conductivity, slip length

## Abstract

In this paper, we propose an alternative road to calculate the transport coefficients of fluids and the slip length inside nano-conduits in a Poiseuille-like geometry. These are all computationally demanding properties that depend on dynamic, thermal, and geometrical characteristics of the implied fluid and the wall material. By introducing the genetic programming-based method of symbolic regression, we are able to derive interpretable data-based mathematical expressions based on previous molecular dynamics simulation data. Emphasis is placed on the physical interpretability of the symbolic expressions. The outcome is a set of mathematical equations, with reduced complexity and increased accuracy, that adhere to existing domain knowledge and can be exploited in fluid property interpolation and extrapolation, bypassing timely simulations when possible.

## 1. Introduction

The utilization of machine learning (ML) techniques in current physics and engineering problems is anticipated to expand across all fields that involve numerical data. The idea of creating new knowledge via predictions based on historical simulation or experimental data is recently trending. Modern computational approaches try to bind techniques with traditional frameworks, taking advantage of their adaptability and ability to provide faster and, oftentimes, accurate predictions in scientific and technological applications. To mention a few, ML-assisted frameworks have been incorporated for atomic-scale force field extraction [1], fluid flow estimation [2], materials science [3,4,5], construction [6,7], energy [8,9], and chemical industry applications [10,11,12].

A major challenge that drives the evolution of new and existing ML algorithms is the transparency of the methods employed and interpretability of the results. Towards this perspective, symbolic regression (SR) methods have emerged. Symbolic regression is an ML method that derives mathematical equations in closed form based on available data using Genetic Programming (GP) principles, without prior knowledge of the system under investigation. A pool of candidate equations is stochastically extracted through crossover and mutation operations [13] and the user has to select those that best fit the physical problem. Unlike black-box models, SR allows us to uncover the underlying mechanisms of the studied system, clarify ambiguous relationships between variables, and provide a more physical understanding [14].

In such data-driven approaches, the main factor that determines the performance of each model is based upon the quality and quantity of the input dataset. Keeping in mind that oftentimes experimental measurements are hard to obtain, the common data acquisition method is the utilization of simulation or synthetic data to train an ML model. Since the presence of walls within nano-conduits significantly affects the system, continuum theory is not applicable, and the calculation of transport coefficients becomes ambiguous, making equilibrium methods insufficient [15]. The macroscale approach to fluid dynamics is mainly based on the Navier–Stokes (NS) equations. However, the fundamental hypothesis of the no-slip condition fails to anticipate phenomena occurring at the micro- and nano-scale [16].

In bulk systems, static and dynamic property calculation is carried out at the micro- and nano- scale with relations usually coming from statistical mechanics. In nano- and macro-devices, confinement between solid surfaces, the implied boundary conditions, as well as the interaction between fluid and wall atoms lead to fundamentally different mechanics of their mass and energy transport [17], affect fluid properties [18], and make their estimation harder [19]. Molecular dynamics (MD) simulations seem to be the most prominent solution for their investigation, which involves calculating particle positions under a given potential, incorporating Newton’s second law [20,21,22,23,24]. This characteristic allows the calculation of transport phenomena accurately via equilibrium MD (EMD) and non-equilibrium MD (NEMD) frameworks.

Due to the complex nature of the investigated system, e.g., geometrical discontinuities, molecule adsorption on walls, or even the extension of the wall force field inside neighboring fluid layers [25], EMD approaches are less applicable and NEMD has to be employed [26]. Nevertheless, both in EMD and NEMD, the calculation of the diffusion coefficient, shear viscosity, and thermal conductivity requires complex relationships, such as the Green–Kubo (GK) equations, which are computationally intensive and rely on expensive experimental procedures [27]. This complex nanochannel environment is further amplified by the breakdown of the no-slip condition [19], which gives rise to a fluid/surface property known as the slip length [28,29]. This has an impact on material surface properties and the rate of mass flow. Specifically, surfaces that have been engineered to achieve specific properties can generate desirable slip lengths to control flow rates in various applications.

In this paper, we propose an alternative approach that relies on data derived from MD simulations. In the sections that follow, we discuss conventional computational approaches, present MD simulation details, and argue for SR applicability (Section 2). Furthermore, we present the generated analytical expressions and compare their accuracy characteristics (Section 3), dive deep into the physics hidden behind the obtained mathematical operators (Section 4), and lastly, propose directions for further research that could be incorporated in designing micro-devices at small scales (Section 5).

## 2. Computational Methods

Next, we present all theoretical relations and parameters employed in the simulation model, as well as the SR techniques and methods incorporated to harness the dataset.

### 2.1. Mathematical Relations

#### 2.1.1. Particle Interaction Potentials

A well-established particle interaction potential for MD simulations is the Lennard–Jones 12-6 (LJ) potential, $u_{r_{ij}}^{LJ}$, defined between the *i* and *j* particles as [30]

(1) $$u_{r_{ij}}^{LJ}=\{\begin{array}{cc} 4\epsilon \left[{\left(\frac{\sigma }{r_{ij}}\right)}^{12}-{\left(\frac{\sigma }{r_{ij}}\right)}^{6}\right], & r_{ij}<r_{c}\\ 0, & r_{ij}\geq r_{c}>\sigma \end{array}$$

where $r_{ij}$ denotes the *i* and *j* particle distance, ϵ and σ are model energy and size parameters, respectively, and $r_{c}$ is the cutoff radius. The LJ potential is capable of modeling bulk or multi-particle systems and ϵ and σ parameters are computed accordingly (see Ref. [31] for more details). In nanochannel flows, where fluid particles coexist with solid, wall particles, the wall may be considered either as static or as an array of particles that fluctuate around their equilibrium positions by applying an elastic spring potential [32],

(2) $$u_{wall}\left(\left|r\left(t\right)-r_{eq}\right|\right)=\frac{1}{2}K{\left(\left|r\left(t\right)-r_{eq}\right|\right)}^{2},$$

where r(t) refers to the particle’s position at time *t*, $r_{eq}$ is its initial lattice position, and *K* is the spring constant, with a high value of *K* denoting a rigid wall.

In the case of polar liquids, coulombic long-range interactions must also be taken into account, with the electric potential being [32]

(3) $$E=\frac{Cq_{i}q_{j}}{er}\;r<r_{c}\;.$$

Here, $q_{i}$ and $q_{j}$ are atoms’ charges, *C* is the energy conversion constant, and *e* is the dielectric constant.

#### 2.1.2. Transport Properties

The self-diffusion coefficient (D) can be calculated by either the Einstein equation [31],

(4) $$D=\lim_{t\to \infty }\frac{1}{2dNt}\langle \sum_{j=1}^{N}{\left[r_{j}\left(t\right)-r_{j}\left(0\right)\right]}^{2}\rangle ,$$

or the GK relation,

(5) $$D=\frac{1}{3N}\int_{0}^{\infty }\langle \sum_{j=1}^{N}v_{j}\left(0\right)\cdot v_{j}\left(t\right)\rangle ,$$

where $r_{j}$ denotes the $j^{th}$ particle’s position vector and $v_{j}$ its velocity vector, the dimensionality of the system is *d*, and *N* is the total number of particles. The former diffusion coefficient equation is generally incorporated in equilibrium systems; however, by omitting drift from the fluid flow, one could apply them in non-equilibrium configurations as well [33].

Similarly, the GK formalization for thermal conductivity (λ) is given by

(6) $$\lambda =\frac{1}{Vk_{B}T^{2}}\int_{0}^{\infty }dt<J_{q}^{x}\left(t\right)\cdot J_{q}^{x}\left(0\right)>,$$

with $J_{q}$ being the microscopic heat flow

(7) $$J_{q}=\frac{1}{2}\sum_{i=1}^{N}m_{i}{\left(v_{i}\right)}^{2}v_{i}-\sum_{i=1}^{N}\sum_{j>1}^{N}\left[r_{ij}:\frac{\partial u(r_{ij})}{\partial r_{ij}}-I\cdot u\left(r_{ij}\right)\right]\cdot v_{i}$$

where $v_{i}$ is the $i^{th}$ atom’s velocity vector and **I** is the unitary matrix.

Finally, the respective GK equation for shear viscosity, (η), is

(8) $$\eta =\frac{1}{Vk_{B}T}\int_{0}^{\infty }dt<J_{p}^{xy}\left(t\right)\cdot J_{p}^{xy}\left(0\right)>,$$

where the off-diagonal elements of the microscopic stress tensor denoted by $J_{p}^{xy}$ are described by

(9) $$J_{p}^{xy}=\sum_{i=1}^{N}m_{i}v_{i}^{x}v_{i}^{y}-\sum_{i=1}^{N}\sum_{j>1}^{N}r_{ij}^{x}\frac{\partial u(r_{ij})}{\partial r_{ij}^{y}}\;,$$

with $u(r_{ij})$ indicating the LJ potential of Equation (1); the distance between *i* and *j* particles is $r_{i,j}$, and $v_{i}^{j}$ is the $i^{th}$ particle velocity for j=x,y,z directions. We have to note that thermal conductivity (Equation (6)) and shear viscosity (Equation (8)) are derived only for equilibrium conditions or, at least, for systems close to equilibrium [34].

#### 2.1.3. Slip Length

**Figure 1:**
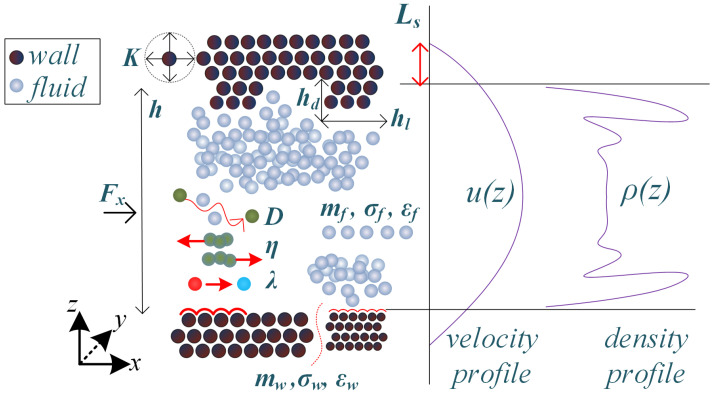
Molecular dynamics model, with all parameters involved in flows between two infinite plates, as shown in Table 1 and Table 2. The mechanisms of diffusion, *D*, viscosity, η, thermal conductivity, λ, and the slip length, $L_{s}$, are abstractly presented, along with a characteristic velocity and density profile at the nanoscale.

Fluid confinement investigation requires proper designing of the modeling system, as density appears to be non-homogeneous at the wall’s vicinity [35] due to wall characteristics (e.g., degree of wettability and mass of each particle) [36], topology of the surface, and thermal, atomic, or geometrical roughness [37]. As already mentioned, in the macroscale hypothesis of no-slip breakdown [28,38], the slip length $\left(L_{s}\right)$ has to be taken into account, from the following equation [32]:

(10) $$L_{s}=u_{w}/\frac{du_{w,z}}{dz}{|}_{w},$$

with $u_{w}$ being fluid velocity at the wall. In a nanochannel of height *h*, the dimensionless slip length $L_{s}^{*}$, is given by the slip length-to-channel height ratio $L_{s}/h$. Its values can be calculated by projecting the fluid’s velocity profile until the point where it vanishes inside the wall, as shown in Figure 1.

### 2.2. Simulation Model and Dataset Creation

A Poiseuille-based simulation model is considered for dataset creation [39]. More specifically, a monatomic LJ liquid flows between two infinite, solid plates, in various cases of flat or grooved walls (Figure 1). Next, we refer to fluid quantities with index *f* and *w* for walls. The distance between the two plates in the *z*-direction is *h*, groove height and length are depicted as $h_{g}$ and $h_{l}$, respectively, while periodic boundary conditions are set in *x*- and *y*-directions. A cutoff radius equal to $r_{c}=2.5\sigma$ has been considered for the LJ potential (Equation (1)), while ϵ and σ resemble those of argon (Ar), i.e, $\sigma_{f}=\sigma_{w}=0.3405$ nm, $\epsilon_{f}/k_{B}=119.8$ K ($k_{B}$: Boltzmann constant). The particle mass has been set equal to $m_{Ar}=39.95\;a.u.$.

To account for various surface wettability properties, the ratio $\epsilon_{wf}/\epsilon_{f\;f}$ corresponds to “hydrophobic” walls when $\epsilon_{wf}/\epsilon_{f\;f}$ is close to zero and "hydrophilic" walls when $\epsilon_{wf}/\epsilon_{f\;f}$ approaches unity (see Ref. [40] for details). Fluid particles flow due to an external force $F_{ext}$ applied to every fluid particle, small enough to remain close to the linear regime [41,42]. The application of Nosé–Hoover thermostats at the walls [43,44] keeps the system at the NVT ensemble, with constant temperature. To achieve enhanced thermalization of the wall particles, two distinct thermostats have been used, one for the upper wall and another for the lower wall. The processes of self-diffusion, viscosity, and thermal conductivity, as well as the slip length, are briefly summarized in Figure 1. These properties are affected by confinement, which leads to fluid particle ordering near the walls (as shown in the density profile in Figure 1).

All parameters of the MD simulation, along with the derived transport properties and the slip length, are given in Table 1 and Table 2.

**Table 1:** Transport property dataset value range.

Parameters	Diffusion Coefficient	Shear Viscosity	Thermal Conductivity
Channel height (h)	2.64–100.44	2.64–100.44	2.64–100.44
External force $\left(F_{ext}\right)$	0.0004–0.0743	0.0004–0.0743	0.0004–0.0743
Energy ratios $\left(\epsilon_{wf}/\epsilon_{f\;f}\right)$	0.1–5.0	0.1–5.0	0.1–5.0
Transport property $\left(D_{c},\;\eta_{c},\;\lambda_{c}\right)$	1.227–10.3575	0.7479–3.1946	1.73–3.1163
Number of observations	54	54	54

**Table 2:** Slip length dataset value range.

Parameters	Min	Max
Channel height (h)	1.049869	210.0
Groove length to channel height $\left(h_{l}/h\right)$	0.0119	1.0
Groove height to channel height $\left(h_{d}/h\right)$	0.0	2.05
Wall-to-fluid energy interaction ratio $\left(\epsilon_{wf}/\epsilon_{f\;f}\right)$	0.1	2.236
Wall-to-fluid particle size ratio $\left(\sigma_{wf}/\sigma_{f\;f}\right)$	1.0	3.0
Wall-to-fluid particle mass ratio $\left(m_{w}/m_{f}\right)$	0.663	20.0
External force $\left(F_{ext}\right)$	0.0	4.9
Wall spring constant $\left(K^{*}\right)$	57.15	10,000.0
Reduced temperature $\left(T^{*}\right)$	0.8333	2.59
Reduced density $\left(\rho^{*}\right)$	0.0468	1.303
Slip length-to-channel height ratio $\left(L_{s}/h\right)$	0.0	7.677928
Number of observations		343

### 2.3. Symbolic Regression

Symbolic regression is a supervised ML algorithm that constructs a mathematical function based on a given dataset. It is differentiated by other regression instances (e.g., linear, multivariate, polynomial, etc.) as both structure and constitutive parts of the expression are being selected on the fly. This feature diminishes the need to include some kind of prior knowledge into the procedure and allows a completely data-driven evolution. In contrast to the traditional black-box ML models (e.g., neural networks), SR presents a clearer linkage between the system’s variables and, at the same time, reveals hidden dynamics.

**Figure 2:**
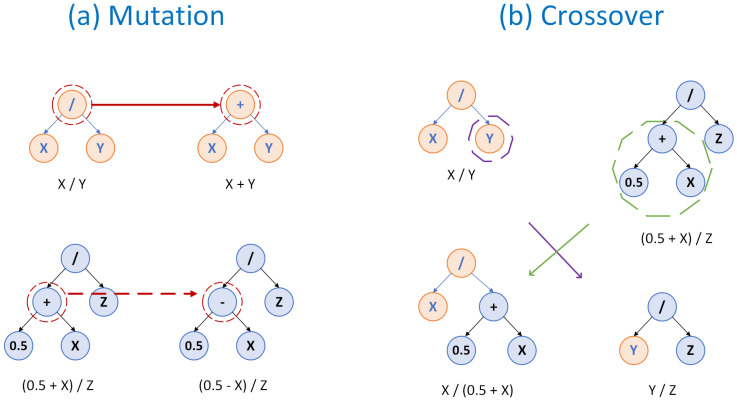
The processes of (**a**) mutation and (**b**) crossover in genetic programming, shown in a tree structure form. In mutation, new equation nodes or branches can substitute less accurate ones, while in crossover, branches can be swapped between different trees.

The common SR implementation is based on GP principles, an Evolutionary Algorithm (EA) subset that approaches the optimal solution systematically in a procedure that resembles Darwin’s theory of evolution. First, a pre-defined number of random expressions is created in a large set (population), where each equation (individual) can be visualized as a tree (see Figure 2). Secondly, the accuracy of each individual is estimated by a measure of fitness, such as the Mean Squared Error (MSE, Equation (11)). Then, "strong" equations that achieve small errors are tabulated and reproduced in a subsequent population by employing the GP operations of crossover and mutation (see Figure 2). Step after step, more robust expressions are extracted, and finally, several expressions are exported by balancing accuracy and complexity characteristics (Pareto frontier). The complexity term that usually accounts for the number of nodes of the tree structure can facilitate a general understanding of the given network. More details on the method can be found in [14,45].

(11) $$MSE=\frac{1}{n}\sum_{i=1}^{n}{\left(Y_{i}-\hat{Y_{i}}\right)}^{2}\;,$$

The application of SR in current physical science problems is gaining ground [14], opening a new route to re-evaluate traditional empirical and approximate equations. Examples include the prediction of fluid properties [11,46,47,48], fatigue life [49,50], modeling plastic deformation [51], and data-driven proof of physical and chemical laws [52], among others.

## 3. Results

Four distinct datasets that provide the self-diffusion coefficient (D), thermal conductivity (λ), shear viscosity η, and slip length $\left(L_{s}\right)$ are considered, created by MD simulations and literature review (Table 1 and Table 2). An SR framework based on PySR [53,54] and our own Python code follows to obtain a pool of candidate equations. Since finding an appropriate expression is a multi-objective optimization problem, more than one optimal solution is produced.

For the selection of final expressions, several factors have been taken into consideration. At first, since the establishment of a direct linkage between the MD structure variables in current theoretical approaches is scarce, we focused on repeating forms at the generated expressions. Moreover, as the formation of an equation via GP principles is a stochastic procedure, an intermittent re-appearance of several configurations could be an indication of capturing part of the system’s dynamics. Secondly, their ability to physically describe the system and their inherent complexity (Compl.) was taken into account. The final criterion was their prediction accuracy, as revealed by measures such as the R-squared $\left(R^{2}\right)$, mean absolute error (MAE), (MSE), and root MSE (RMSE) values.

After a significant number of parallel SR calculations, the dominant equations have been selected for the self-diffusion coefficient $\left(D_{c}^{*}\right)$, thermal conductivity $\left(\lambda_{c}^{*}\right)$, shear viscosity $\left(\eta_{c}^{*}\right)$, and slip length $\left(L_{s}^{*}\right)$, as follows:

(12) $$D_{c}^{*}=\sqrt{h+2\log(h)-2\log\left(\epsilon_{wf}/\epsilon_{f\;f}\right)}-\sqrt{F_{ext}\;h}\;,$$

(13) $$\lambda_{c}^{*}=log\left(F_{ext}\;h+u_{1}\sqrt{u_{2}h+e^{\epsilon_{wf}/\epsilon_{f\;f}}}+u_{3}\right)\;,$$

(14) $$\eta_{c}^{*}={\left[e^{\epsilon_{wf}/\epsilon_{f\;f}\left(-u_{1}\;F_{ext}+1\right)}e^{u_{2}}\right]}^{\frac{1}{h}}\;,$$

(15) $$L_{s}^{*}=\frac{w_{1}\left(h_{l}/h\right)+w_{2}{\left(m_{w}/m_{f}\right)}^{2}e^{-w_{3}{\left(\epsilon_{wf}/\epsilon_{f\;f}\right)}^{4}}+w_{4}\sqrt{\left(h_{d}/h\right)}}{h^{2}}\;,$$

where $u_{1}=1.6,\;u_{2}=0.39,\;u_{3}=2.47,v_{1}=0.732,\;v_{2}=0.882,w_{1}=0.13,\;w_{2}=1.3,$ $w_{3}=w_{4}=4$.

**Figure 3:**
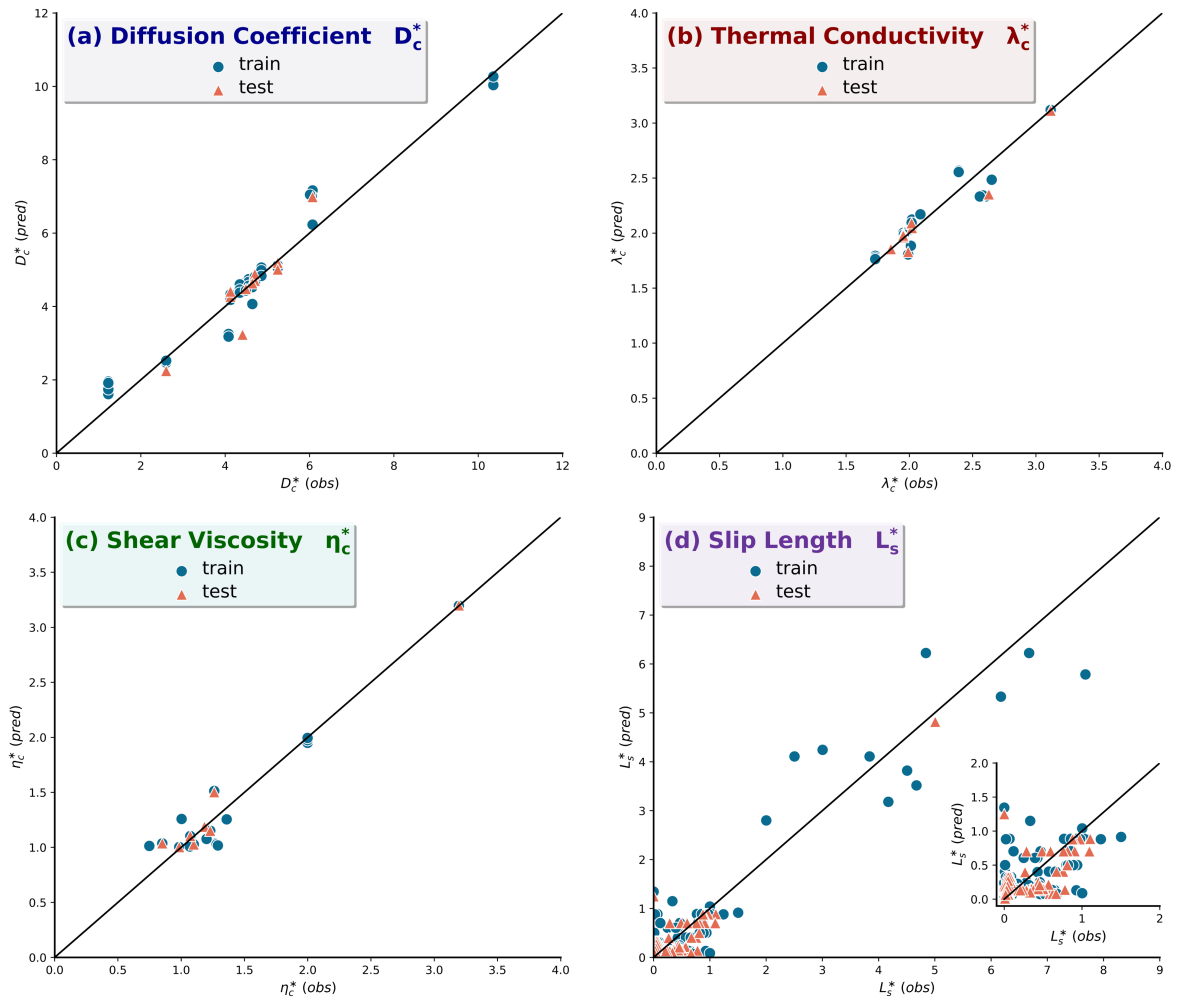
Identity plots for the generated expressions for (**a**) $D_{c}^{*}$, (**b**) $\;\lambda_{c}^{*}$, (**c**) $\eta_{c}^{*}$, and (**d**) $L_{s}^{*}$. The 45° line is a guide to the eye, denoting perfect prediction when data points lie on it.

Although the application of the SR algorithm in small datasets [55] may lead to over-fitting, the above equations achieve relatively high accuracy scores. The identity plots for the generated expressions and a zoomed figure of the dense regions can be found in Figure 3. In addition, their complexity values and overall accuracy across several random states, setting a partitioning factor of 70% train and 30% test, are shown in Table 3.

It should be noted that $D_{c}^{*}$, $\lambda_{c}^{*}$, and $\eta_{c}^{*}$ final expressions (Equations (12)–(14)) incorporate every input parameter of the dataset. In contrast, the slip length formula $L_{s}^{*}$ (Equation (15)) spots only the important parameters and ends up with the wall roughness parameters $h_{l}/h$ and $h_{d}/h$, wall-to-fluid interaction and mass ratio $\epsilon_{wf}/\epsilon_{f\;f}$ and $m_{w}/m_{f}$, respectively, and the channel height *h*. The slip length equation reports a complexity value of Compl=19. One could opt for different equations than those presented with more or less advanced complexity; however, a trade-off between complexity and accuracy has to be retained [14].

From a statistical point of view, transport property expressions achieve fine accuracy. This is further supported by minor residual errors observed in the respective identity plots (Figure 3). On the contrary, slip length accuracy measures are smaller. A point worth mentioning is the fact that training and testing accuracy values are balanced, and this is evidence of the absence of over-fitting. In other words, the SR algorithm trained on the available data may be incorporated for predictions outside (extrapolation) and between (interpolation) the available data points, making this framework both transparent and generalizable.

## 4. Discussion

The inherent computational complexity of MD simulations is mainly focused on calculating the interactions between the system’s particles. These interactions are typically calculated for millions of particles, for every time step (in the order of fs) considered. On the other hand, the proposed ML framework can provide an alternative to conventional research methods by approaching a possible solution at only a fraction of the initial computational demand. However, first we have to ensure that the proposed equations are closely connected to the physics of the investigating system. The generated expressions are purely data-driven and able to provide a direct estimation of the targeted quantity (i.e., the three transport properties $D_{c}^{*}$, $\lambda_{c}^{*}$, and $\eta_{c}^{*}$ and the slip length $L_{s}^{*}$) as functions of the atomic-scale parameters of the confined system.

Equation (12) for $D_{c}^{*}$ presents a strong dependence on channel height, *h*. This trend agrees with simulation results [31], where it has been shown that the self-diffusion coefficient decreases in nanochannels of small width and approaches its bulk value in wider channels, where the wall effect is minimum. It is also observed that a square root relation between $D_{c}^{*}$, $\epsilon_{wf}/\epsilon_{f\;f}$, and $F_{ext}$ exists; however, it is difficult to decompose their effect on the self-diffusion coefficient. It seems that $D_{c}^{*}$ is affected by $\epsilon_{wf}/\epsilon_{f\;f}$ in small channel heights.

On the other hand, the physical explanation is clearer for thermal conductivity, as $\lambda_{c}^{*}$ in Equation (13) seems to be a logarithmic function, where *h*, $F_{ext}$, and $\epsilon_{wf}/\epsilon_{f\;f}$ positively affect its value. As far as shear viscosity is concerned, Equation (14) presents a complex exponential behavior, where $\eta_{c}^{*}$ is affected mainly by $\epsilon_{wf}/\epsilon_{f\;f}$ and *h*. Here, as h→0, shear viscosity increases, for a given $\epsilon_{wf}/\epsilon_{f\;f}$. Moreover, an increase in $\epsilon_{wf}/\epsilon_{f\;f}$ leads to an increase in $\eta_{c}^{*}$. The external force in most of our simulation cases lies in the range $0.01\leq F_{ext}\leq 0.5$ (some extreme values have been used only for testing), which means that the term $u_{1}\;F_{ext}+1>0$ only slightly decreases $\eta_{c}^{*}$, especially in narrower channels (small *h*).

**Figure 4:**
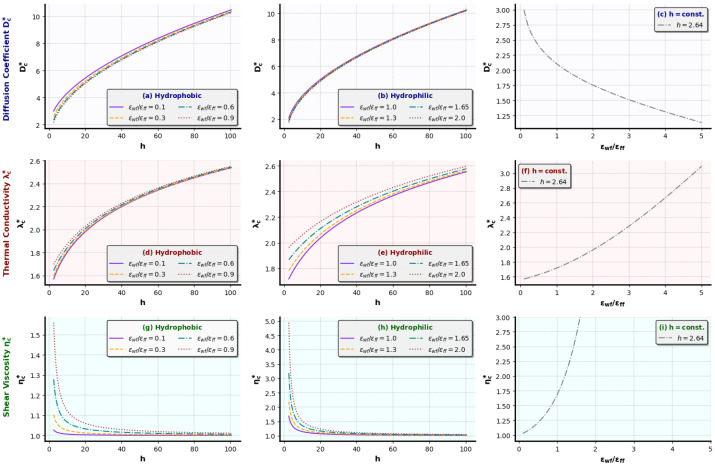
Examining the behavior of the proposed SR equations for $D_{c}^{*}$ vs. *h* in (**a**) hydrophobic and (**b**) hydrophilic channels, and (**c**) $D_{c}^{*}$ vs. $\epsilon_{wf}/\epsilon_{f\;f}$ for h=2.64σ, for $\lambda_{c}^{*}$ vs. *h* in (**d**) hydrophobic and (**e**) hydrophilic channels and (**f**) $\lambda_{c}^{*}$ vs. $\epsilon_{wf}/\epsilon_{f\;f}$ for h=2.64σ, and for $\eta_{c}^{*}$ vs. *h* in (**g**) hydrophobic and (**h**) hydrophilic channels and (**i**) $\eta_{c}^{*}$ vs. $\epsilon_{wf}/\epsilon_{f\;f}$ for h=2.64σ.

In order to obtain a clear view of each input parameter effect on transport properties in confined channels, we provide a quantified evaluation of the SR expressions’ responses in different scenarios in Figure 4. Here we have omitted the effect of the external force, as it has been found that it does not affect the outcome significantly, since its effect on flow is subtracted from the final calculations in GK approaches (see Ref. [31] for more details), and argue for the effect of $\epsilon_{wf}/\epsilon_{f\;f}$ and *h*.

The proposed SR-derived equation for the self-diffusion coefficient in hydrophobic and hydrophilic nanochannels (Figure 4a,b, respectively) presents a clear increasing behavior as *h* increases. In narrower channels, the effect of $\epsilon_{wf}/\epsilon_{f\;f}$ becomes significant. Hydrophobic nanochannel walls ($\epsilon_{wf}/\epsilon_{f\;f}\to$ 0) lead to higher self-diffusion values compared to hydrophilic ones ($\epsilon_{wf}/\epsilon_{f\;f}$ > 1). This illustrates the fact that, in hydrophilic cases, where particles are attracted to the surface, fluid particles find it difficult to diffuse. On the contrary, in a hydrophobic case, where atoms are repelled from the wall surface, fluid diffusion is facilitated. It is also shown that, for a nanochannel of constant height, e.g., h=2.64σ, where the wall effect is strong due to the small height, $D_{c}^{*}$ decreases as the walls become more hydrophilic (Figure 4c).

Another important remark is that shear viscosity from the SR-derived Equation (14) for both hydrophobic and hydrophilic channels, shown in Figure 4g,h, respectively, clearly obtains its bulk value for h>20σ. This is in fine agreement with our previous numerical investigation in [15]. Larger $\eta_{c}^{*}$ values are obtained for hydrophilic walls, and this is also shown in Figure 4i, for the case of h=2.64σ. Furthermore, thermal conductivity SR Equation (13) reveals a $\lambda_{c}^{*}$ increase vs. $\epsilon_{wf}/\epsilon_{f\;f}$ and *h*, in Figure 4d,e. Higher $\lambda_{c}^{*}$ values refer to more hydrophilic walls. It is worth noting that thermal conductivity inside narrow channels, i.e., for h=2.64σ, increases as the walls become more hydrophilic (Figure 4f).

In case of slip length prediction from the proposed Equation (15), we observe that $L_{s}^{*}$ is inversely proportional to the squared channel height, $h^{2}$. This is physically correct, since in channels of large heights, the no-slip condition applies [19]. Moreover, geometrical parameters of the walls ($h_{l}/h,h_{d}/h$) and particle masses $\left(m_{w}/m_{f}\right)$ seem to have a positive effect on the outcome. We attribute this to the fact that rough nanochannels have been widely incorporated to achieve larger slip lengths at the nanoscale [37,39,56]. Conversely, the energy ratio $\epsilon_{wf}/\epsilon_{f\;f}$ has a negative exponential effect on $L_{s}^{*}$. This is also an expected behavior, since as $\epsilon_{wf}/\epsilon_{f\;f}$ increases, the walls become more hydrophilic, imposing difficulties in fluid movement, and slip length decreases.

**Figure 5:**
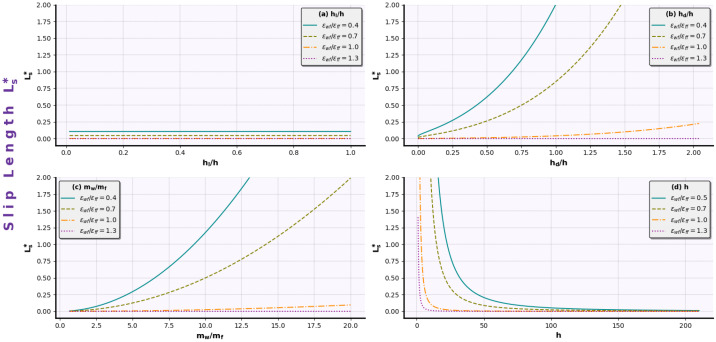
Slip length equation dynamics for various $\epsilon_{wf}/\epsilon_{f\;f}$ vs. (**a**) $h_{l}/h$, (**b**) $h_{d}/h$, (**c**) $m_{w}/m_{f}$, and (**d**) *h*.

Slip length behavior is graphically presented in Figure 5. Each one of the independent variables has been held constant at the mean value ($\bar{h_{l}/h}=0.7,\;\bar{h_{d}/h}=0.07,\;\bar{m_{w}/m_{f}}=3.0,$ $\bar{h}=16.96$) and the effect of one variable is depicted in the respective sub-figure. Firstly, we observe that groove length has a minor influence on the outcome (Figure 5a), since slip length values remain constant when increasing the $h_{l}/h$ ratio. On the other hand, slip length is highly affected by the depth of the groove (Figure 5b). Apparently, the SR procedure has identified that groove depth overwhelms the groove length impact, especially when wall/fluid interactions constitute a super hydrophobic environment. This can also be seen by the proposed equation weights on each of the corresponding variables in Equation (15), where the groove depth weight ($w_{4}$) is sufficiently larger than the weight of groove length ($w_{1}$).

Similarly, the slip length increases as the wall/fluid particle mass ratio $m_{w}/m_{f}$ increases (Figure 5c). Larger wall particle masses compared to fluid particles would pose an atomic roughness wall, which, along with strong hydrophobic interactions, increases the slip length. As far as the slip length and channel width, *h*, are concerned (Figure 5d), the former assumption of the inverse proportionality is evident and, ultimately, leads to the restoration of the no-slip assumption as bulk behavior is approached.

A common characteristic in every subfigure in Figure 5 is that the wall-to-fluid energy interaction ratio $\left(\epsilon_{wf}/\epsilon_{f\;f}\right)$ has a major impact at the nanoscale, as far as the slip length is concerned. For hydrophobic cases where fluid atoms become subject to higher repulsion by the wall atoms, a large slip length value can be expected due to the reduction in the friction between fluid and wall as a result of the wall–fluid interaction area minimization [57]. Conclusively, the proposed Equation (15), derived from the data-driven SR method, seems to capture the physical interpretation of the slip mechanism in confined flows.

## 5. Conclusions

Recently, data-based methodologies have progressively entered and enhanced conventional approaches by concurrently establishing computational efficiency and economy. Toward this end, an ML architecture has been trained on data derived from NEMD simulations and found broad applicability in the construction of micro- and nano-devices, i.e., the self-diffusion coefficient, thermal conductivity, shear viscosity, and the slip length. The outcome is a set of closed form equations that perform fine in several cases investigated and are able to decode hidden mechanics, even with no prior understanding of the system.

The SR-based self-diffusion coefficient equation has identified the tendency of fluid particles to stick onto hydrophilic channel walls and achieves lower values as the wall-to-fluid interaction ratio increases. In the same way, thermal conductivity and viscosity equations are increased inside hydrophilic nanochannels, especially in narrow channels. On the other hand, the suggested slip length equation applies only in channels of small height, *h*, and agrees with the macroscale hypothesis of the no-slip condition for h>20σ.

We believe that symbolic regression techniques have matured and new computational approaches will emerge that are able to bind these data-driven methods to classical simulations at all scales. Special emphasis should be placed on the physical explanation of system dynamics, which, in this paper, has been found to agree with the established domain knowledge. The area of fluids under confinement investigation and fluid-to-surface interaction dynamics present fundamentally different behavior than the bulk, and novel, interpretable machine learning techniques could aid traditional approaches in addressing current computational obstacles.

## Figures and Tables

**Table 3:** Generated expressions specifications.

Equation		$R^{2}$		MSE		RMSE		MAE		Compl.
		Train	Test	Train	Test	Train	Test	Train	Test	
$D_{c}^{*}$ (Equation (12))		0.94	0.91	0.18	0.17	0.42	0.41	0.28	0.27	15
$\lambda_{c}^{*}$ (Equation (13))		0.92	0.90	0.01	0.01	0.10	0.10	0.07	0.07	14
$\eta_{c}^{*}$ (Equation (14))		0.95	0.93	0.02	0.02	0.14	0.13	0.10	0.10	12
$L_{s}^{*}$ (Equation (15))		0.86	0.83	0.11	0.11	0.33	0.33	0.20	0.21	19

## Data Availability

Data may be available from the corresponding author upon reasonable request.
